# ATP13A2 as a prognostic biomarker and its correlation with immune infiltration in cervical cancer: A retrospective study

**DOI:** 10.1111/jcmm.70097

**Published:** 2025-04-08

**Authors:** Zhi Zhao, Yijie Peng, Yuanyuan Yang, Shuaiyu Li, Jiang Ling, Zhenyu Zhu, Chenfeng He

**Affiliations:** ^1^ Zhengzhou Yihe Hospital, Postdoctoral Innovation Practice Base Henan University Zhengzhou Henan China; ^2^ Department of Breast Surgery Graduate School of Medicine, Kyoto University Kyoto Japan; ^3^ Department of Hepatobiliary and Pancreatic Surgery The Central Hospital of Shaoyang Shaoyang Hunan China; ^4^ Clinical Research Center for Women's Reproductive Health in Hunan Province Changsha Hunan China; ^5^ Reproductive Medicine Center Xiangya Hospital of Central South University Changsha China; ^6^ School of Information Science Kyushu University Fukuoka Japan; ^7^ Department of Forensic Science, School of Basic Medical Sciences Central South University Changsha Hunan China; ^8^ Department of Integrative Bioanalytics Institute of Development, Aging and Cancer (IDAC), Tohoku University Sendai Japan

**Keywords:** ATP13A2, cervical cancer, immune infiltration, microbiome, prognostic marker

## Abstract

While the oncogene ATP13A2 is reportedly involved in colorectal cancer, its role in cervical cancer (CC) has yet to be fully characterized. In this study, we investigated ATP13A2 as a potential prognostic biomarker of CC. To this end, we compared CC tissues with normal tissues to identify differentially expressed genes, identifying ATP13A2 as a potential marker of CC. Elevated ATP13A2 expression levels were identified in CC samples compared to noncancerous samples across various data sets, with further immunohistochemical validation. Functional enrichment analysis revealed that ATP13A2 plays an essential role in the CXCL12‐activated CXCR4 signalling pathway and chemotaxis regulation, which may alter immune infiltration. Notably, increased ATP13A2 levels were associated with poor overall survival. Furthermore, multiple clinical characteristics were significantly associated with ATP13A2 expression. Additionally, tumour bacterial infiltration was assessed using weighted co‐expression network analysis, revealing a relationship between ATP13A2 expression and bacteria in the CC tumour microenvironment. Our results suggest that ATP13A2 is a promising diagnostic and prognostic marker for CC. However, further large‐scale studies are needed to fully elucidate the mechanisms underlying the involvement of ATP13A2 in CC.

## 1 INTRODUCTION

Cervical cancer (CC) is the fourth most prevalent cancer among women worldwide and the seventh most common cancer in the general population, with 569,847 new cases diagnosed in 2018^1^ and an estimated increase to 660,000 new cases in 2022 across 185 countries.^2^ The primary pathogenic cause of CC is persistent infection with human papillomavirus (HPV); however, several other risk factors have been described, including smoking and human immunodeficiency virus infection.^3^ CC is caused by abnormal cellular growth in the cervix, which is partially attributed to the integration of viral DNA into the host cellular genome, leading to disrupted gene regulation, oncogene activation, and tumour suppressor gene deactivation.^3^

Accumulating evidence supports a significant role for the tumour‐immune microenvironment (TIME) in cancer progression and patient prognosis.^4^, ^5^ The TIME influences cancer growth, invasion, metastasis, drug resistance, and immune escape.^6^ For example, the TIME promotes the occurrence of hepatocellular carcinoma (HCC) through immune escape mechanisms involving natural killer cells and dendritic cells, angiogenesis and tissue remodelling involving macrophages, and the production of cytokines and chemokines by myriad cells.^7^, ^8^ Therefore, targeting TIME modulation may represent an effective strategy for cancer treatment.^9^

Previously, tumour heterogeneity was attributed only to intrinsic genetic changes in cancer cells during proliferation and expansion.^10^ However, recent insights have positioned intratumoral microbiota as a key player in shaping the tumour microenvironment (TME). Intratumoral microbiota is a general term referring to bacteria infiltrating surrounding tissues in the tumour growth environment. Genomics‐based studies have revealed the presence of microbiota in most human cancers.^11^ Remarkably, these microbial communities vary depending on the cancer type, with specific bacteria associated with cancer initiation and progression, influencing therapeutic outcomes and patient survival.^12^, ^13^, ^14^, ^15^ However, the mechanisms by which intratumoral microbiota impact tumour occurrence and progression have not been fully elucidated.^14^, ^15^ Nevertheless, intracellular bacteria can induce gene signatures associated with cancer cell invasion, metastasis, DNA damage repair and cell dormancy in colorectal cancer (CRC). These invasive bacteria recruit myeloid cells, initiating an inflammatory cascade that facilitates T‐cell exclusion and subsequent tumour expansion, regulated by various interleukins and chemokines.^15^

ATP13A2 is a lysosome‐related transmembrane P5‐type ATP transportase with a regulatory role in autophagy, particularly within the context of Parkinson's disease.^16^ However, many studies have also reported abnormal expression patterns of ATP13A2 in various cancers, notably colorectal and hepatic cancers. In particular, correlations have been described between ATP13A2 and cancer progression, metastasis and immune infiltration in HCC cells.^17^ Meanwhile, the absence of ATP13A2 suppresses CRC growth and pentose phosphate pathway (PPP) activity, resulting in decreased pathway products and increased levels of reactive oxygen species.^18^ Although sequence analyses revealed notable homology between ATP13A3, ATP13A4 and ATP13A2,^19^ increased aberrant N6‐methyladenosine modification of ATP13A3 promotes colorectal tumorigenesis, whereas high ATP13A4 expression positively correlates with progression‐free survival in ovarian cancer patients.^20^, ^21^ Given these similarities, we speculated a crucial role of ATP13A2 in tumour progression. However, no previous studies have reported a link between ATP13A2 and CC.

This study investigates ATP13A2 expression and its correlation with CC prognosis using online public databases, namely, the Gene Expression Omnibus (GEO) and The Cancer Genome Atlas (TCGA). Specifically, we analyse the correlation between intratumoral microbiota and ATP13A2 expression levels in CC using TCGA database. This study provides new avenues for investigating the prognostic value of ATP13A2 in CC and suggests an interrelationship and underlying mechanism between intratumoral microbiota and CC progression. Our findings provide a promising foundation for developing targeted treatments based on TME modulation to improve patient outcomes in CC.

## 2 MATERIALS AND METHODS

### 2.1 Patients and specimens

We collected 30 tumour tissue samples from patients with CC who underwent surgical resection. An additional eight cervicitis samples were collected for comparison via colposcopy‐directed biopsy from women diagnosed with chronic cervicitis. All samples were collected at the Xiangya Hospital, Central South University, between 2018 and 2023. In this retrospective study, no CC patients received antitumor treatment before tissue acquisition. All study participants were histologically confirmed cases of primary CC. This study was approved by the Institutional Review Board of the School of Basic Medical Science, Central South University, China (No. 2023‐KT87); written informed consent was obtained from all patients.

### 2.2 Collection of CC data

CC microarray data were obtained from the GEO database (http://www.ncbi.nlm.nih.gov/geo), specifically from the GSE9750 series (Platform: GPL96). To validate the upregulated mRNA expression of ATP13A2 in CC tissues compared with normal tissues, the UALCAN database (http://ualcan.path.uab.edu/), an interactive web portal for in‐depth gene expression data analysis from TCGA, was used. Analyses of immune infiltration and survival prognosis and correlation analyses between ATP13A2 expression and clinical features, and weighted gene co‐expression network analysis (WGCNA) were conducted using the Cervical Squamous Cell Carcinoma (TCGA, PanCancer Atlas) study available on cBioPortal (https://www.cbioportal.org).

### 2.3 Identification of differentially expressed genes and gene function analyses

The microarray data set was downloaded from the GEO query package using the GEO function with accession number GSE9750. A total of 33 CC tissue samples and 21 normal cervical epithelium samples were included in the analysis to identify differentially expressed genes (DEGs) between CC and normal cervical samples. Genes lacking corresponding gene symbols and those with multiple probe sets were removed separately. The statistical significance criteria for DEGs were set at |log2(fold change)| >1 and *p*‐value <0.05. To visualize DEGs, a volcano plot was generated using the ggplot2 (https://ggplot2.tidyverse.org/index.html).

### 2.4 Correlation and ATP13A2‐related gene enrichment analyses

Protein–protein interaction (PPI) network analyses were performed using the online tool STRING v11.5 (https://version‐11‐5. string‐db. org/) with 648 DEGs upregulated in CC as input. The PPI network was constructed with a confidence level threshold of 0.15 (low confidence) and a maximum number of five interactors. The interaction networks associated with ATP13A2 were also visualized. To gain insights into the functional enrichment of ATP13A2‐related DEGs in CC, gene ontology (GO) analyses and Kyoto Encyclopedia of Genes and Genomes (KEGG) were performed using the online tool DAVID v2023q1 (https://david.ncifcrf.gov/). The top 15 significantly enriched GO terms and five KEGG terms were visualized using bubble plots, generated using the ggplot2 (https://ggplot2.tidyverse.org/index.html).

### 2.5 Immune cell infiltration analyses

Transcriptome and immune infiltration data of CC patients were downloaded from the cBioPortal website (https://www.cbioportal.org/), and the ESTIMATE website (https://bioinformatics.mdanderson.org/estimate/index.html) was used to explore the correlation between ATP13A2 and immune infiltration levels.

### 2.6 Assessment of immune scores in CC samples

Immune signature scores were calculated using single‐sample gene set enrichment analysis (ssGSEA) implemented by a GSVA package (version 1.48.3) in R. Clinical results in tumour–immune interactions were evaluated using the Tumour Immune Estimation Resource (TIMER) database (http://timer.cistrome.org/). We obtained a list of 28 immune cells and the related 782 marker genes from Charoentong et al.^22^ Based on the signature genes of 28 different immune cell types, relative immune cell infiltration levels were quantified from gene expression profiles for each tumour sample. Comparative analysis was conducted to evaluate the immune cell infiltration between CC samples with ATP13A2 high expression versus low expression. *p*‐values were calculated using the Wilcoxon rank‐sum and Spearman's rank correlation tests.

### 2.7 Survival prognosis analyses

Survival data for 294 out of 297 patients with CC were sourced from the cBioPortal (https://www.cbioportal.org/), and survival outcomes were analysed using the GraphPad Prism. A median cut‐off of 50% delineated the patients into two groups: low versus high ATP13A2 expression. Kaplan–Meier curves were generated to illustrate the impact of ATP13A2 expression on patient outcomes, including overall survival (OS) and progression‐free survival (PFS). A log‐rank (Mantel–Cox) test was conducted to evaluate the survival significance of ATP13A2‐related genes identified using the STRING database.

### 2.8 Analyses of CC clinical features

Correlations between ATP13A2 expression and age, aneuploidy score, MSI MANTIS score, MSI SENSOR score, and copy number alterations (CNAs) in CC were examined using data from the cBioPortal website (https://www.cbioportal.org/). Data from 290 cases were available for these clinical features. Pearson's analysis and ordinary one‐way analysis of variance (ANOVA) were employed to analyse these associations.

### 2.9 Weighted co‐expression network analyses

WGCNA explored the relationship between the CC microbiome and clinical variables. Relevant data were sourced from the Cervical Squamous Cell Carcinoma study within TCGA PanCancer Atlas, accessed via cBioPortal (https://www.cbioportal.org). A weighted correlation network was constructed from microbiome data using the WGCNA package in R (version 4.3.0). The optimal soft thresholding power was determined by adhering to the approximate scale‐free topology criterion for a robust network fit. After establishing the adjacency matrix with this selected threshold, the microbial taxa with similar abundance patterns were grouped into modules, each comprising a minimum of five taxa. The central measure of these modules, termed the module eigengene, was computed as the first principal component of each module's abundance matrix to succinctly represent the collective expression profile of the taxa. This facilitated the assessment of module‐trait correlations, pinpointing modules potentially linked to ATP13A2 mRNA expression levels and other clinical traits. We plotted the top 10 microbial taxa from the most significant module correlated with ATP13A2 expression in CC to provide insights into their relevance within the disease context.

### 2.10 Immunohistochemistry (IHC) and scoring

IHC was performed on formalin‐fixed paraffin‐embedded tissue sections. The sections were deparaffinized in xylene, rehydrated using gradient concentrations of ethanol, subjected to autoclave antigen retrieval in ethylenediaminetetraacetic acid at 100°C for 5 min, and incubated in 3% hydrogen peroxide for 10 min to recover endogenous peroxidase activity. Next, the tissue sections were washed with phosphate buffer saline (PBS) and incubated with rabbit ATP13A2 antibody (1:75 in ZLI‐9030 antibody diluent; NB110‐41486SS; Novus Biologicals, USA) at 4°C overnight. The sections were washed with PBS and incubated with horseradish peroxidase‐conjugated goat anti‐rabbit secondary antibody (1:1; PV‐6000; Zhongshan Jinqiao, China) for 40 min. All tissue sections were visualized using a DAB kit (Cat. PV‐6000; ZS‐GB BIO, Zhongshan Jinqiao, China), and the nuclei were counterstained with haematoxylin. The IHC results were independently evaluated by two experienced pathologists blinded to the study protocols.

### 2.11 Statistical analysis and data visualization

Statistical significance was assessed using Student's *t*‐test, Mann–Whitney *U*‐test or Chi‐squared test using the GraphPad Prism software (version 10.0). Unless otherwise indicated, data were presented as the mean ± standard error of the mean (SEM), and *p* < 0.05 was considered statistically significant. The n value, representing the number of biological replicates in all experiments, was indicated in the figure legends. Data were visualized using the R and GraphPad Prism 10.0 software.

## 3 RESULTS

### 3.1 Gene expression analysis and identification of DEGs

**FIGURE 1:**
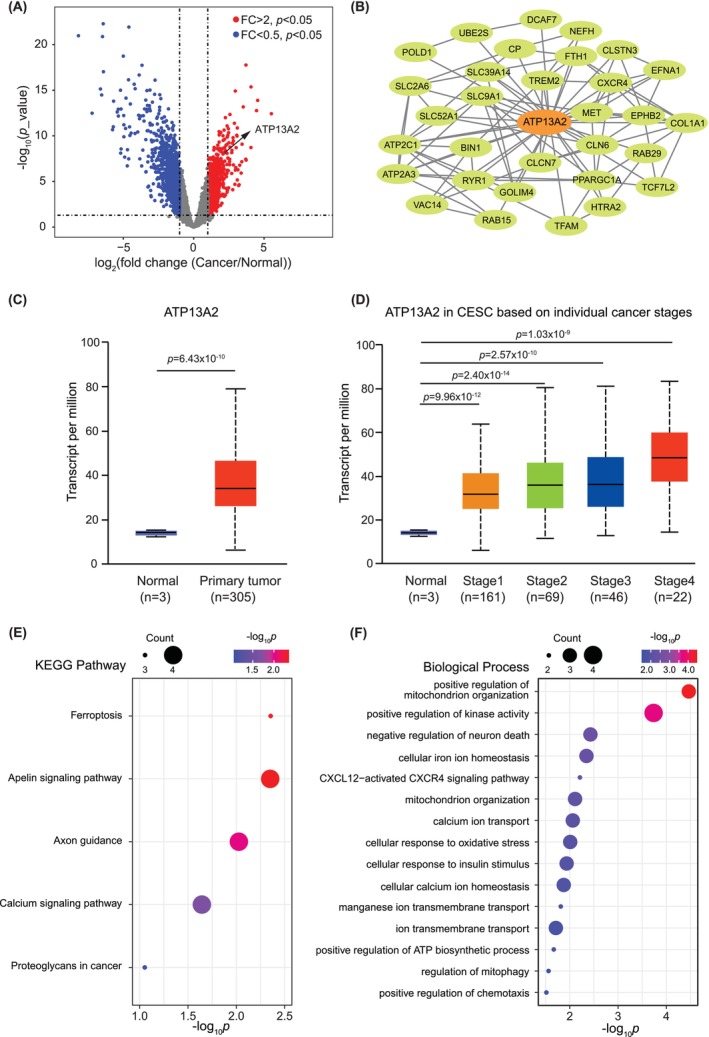
Analysis of cervical cancer‐induced expression and pathway enrichment. (A) Volcano plots representing differential gene expression between cervical cancer and normal tissues using data from the GSE9750 data set. The *x*‐axis displays the log2 (fold change) of gene expression (cancer relative to normal), whereas the *y*‐axis shows the log10 (*p*‐value). Genes with significant upregulation of expression (fold change >2, *p* < 0.05) are highlighted in red, and those with significant downregulation of expression (fold change <0.5, *p* < 0.05) are coloured in blue. ATP13A2 expression is strongly upregulated in cervical cancer and is notably marked. (B) The protein–protein interaction network of cancer‐related genes with upregulated expressions generated using the STRING database emphasized genes related to ATP13A2. A network of ATP13A2‐associated genes is shown. (C) Box plot illustrating the expression levels of ATP13A2 in cervical cancer tissues compared with those in normal tissues. Data are sourced from the UALCAN database, with the *y*‐axis displaying the transcript counts per million. (D) Analysis of ATP13A2 expression across different pathological stages. Data are sourced from the UALCAN database. (E) A representation of the top five terms from the KEGG analysis of genes is shown in (B). The spot size indicates the number of genes in each term, and the colour gradient indicates the *p*‐value of each term. (F) The gene ontology enrichment analysis of gene features in (B). The bubble plot specifies biological processes against their significance (−log10 of the *p*‐value), with the size of each bubble reflecting the number of genes associated with that process.

Using the Limma package, we analysed differences in mRNA expression between CC tissues and normal cervical samples from the GSE9750 dataset. This investigation revealed that ATP13A2 expression was significantly upregulated in cancer tissue samples. Furthermore, 1798 DEGs were highlighted in the volcano plot, of which 648 were upregulated and 1120 were downregulated (Figure 1A).

### 3.2 PPI network construction

To further define the interactions between ATP13A2 and other hub genes, we created a PPI network centred around ATP13A2 based on information from STRING (Figure 1B). The minimum required interactive score was set as low confidence. The resulting ATP13A2‐associated PPI network comprised 31 interacting proteins, including SLC39A14.

### 3.3 Protein expression level analysis

The UALCAN data revealed higher ATP13A2 mRNA expression in primary CC tissues than in normal tissues (Figure 1C). A notable correlation was observed between elevated ATP13A2 expression levels and advanced pathological stages (Figure 1D).

### 3.4 Functional enrichment analysis

The KEGG pathway analysis indicated that ATP13A2‐related genes were primarily enriched in ferroptosis, the apelin signalling pathway, axon guidance and the calcium signalling pathway in cancer (Figure 1E). Furthermore, the GO analysis of biological processes suggested a significant enrichment of ATP13A2‐associated genes in pathways related to the positive regulation of mitochondrial organization, positive regulation of kinase activity, negative regulation of neuronal death, cellular iron ion homeostasis, the CXCL12‐activated CXCR4 signalling pathway and positive regulation of chemotaxis (Figure 1F).

### 3.5 Immunohistochemistry and clinical characteristics

To further investigate the role of ATP13A2 in CC progression, 30 tumour tissue samples and 8 cervicitis samples were compared. The demographic and clinical characteristics of the patients are shown in Table 1. The mean age of CC patients was 53.1 ± 8.6 years, with most presenting with Grade II/III tumours (93.3%). Notably, vessel invasion (63.3%, negative), perineural invasion (90.0%, negative) or lymph node metastasis (90%, negative) was observed in most tumour samples.

**TABLE 1:** Clinical and demographic characteristics of the study population (*n* = 30).

	Mean	SD
Age (years)	53.1	8.6
Invasive depth (mm)	9.3	5.0

	*n*	(%)
Differentiation grade		
1	1	(3.3)
2	18	(60.0)
3	10	(33.3)
NA	1	(3.3)
Vessel invasion		
Negative	19	(63.3)
Positive	11	(36.7)
Perineural invasion		
Negative	27	(90.0)
Positive	3	(10.0)
Lymph node metastasis		
Negative	27	(90.0)
Positive	3	(10.0)
P16		
Weak	22	(73.3)
Moderate	1	(3.3)
Strong	7	(23.3)
ATP13A2		
Negative	6	(20.0)
Weak	5	(16.7)
Moderate	4	(13.3)
Strong	15	(50.0)

Abbreviations: SD, standard deviation; NA, not available.

**FIGURE 2:**
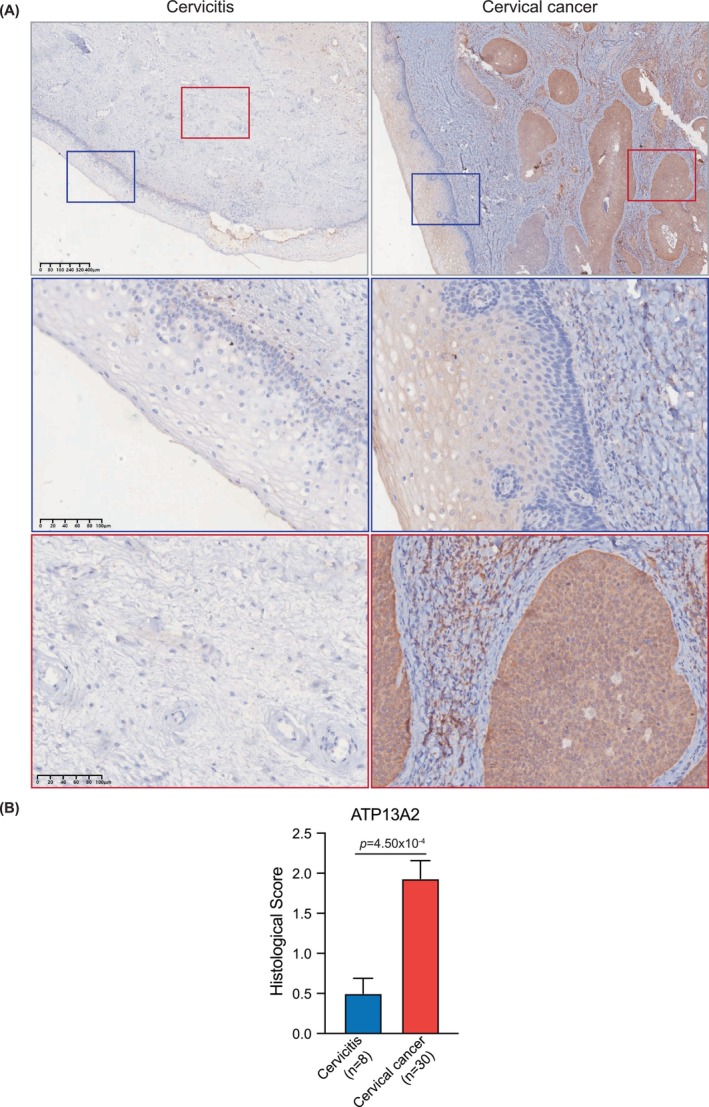
Immunohistochemistry of ATP13A2 antibodies in cervical cancer and cervicitis sections. (A) Representative immunohistochemical staining for ATP13A2 (brown) in cervicitis tissue (left) and human cervical cancer (right) sections. Nuclei were counterstained with haematoxylin (blue staining). The top row displays low‐magnification views (4×), with the areas of interest highlighted by coloured boxes. The corresponding high‐magnification (20×) views of these regions are shown below. The blue and red boxes show the squamous epithelium and stroma, respectively, in the cervical sections. The cervicitis sample showed a negative or weak staining pattern, whereas the cervical cancer sample showed high ATP13A2 protein expression, particularly in the tumour region. (B) Bar plot illustrating the histological scoring of ATP13A2 expression in cervicitis and cervical cancer samples. The data showed a significant increase in ATP13A2 expression in cervical cancer (*n* = 30) compared to that in cervicitis (*n* = 8), with a *p*‐value of 4.50e‐4. The error bars represent the standard error of the mean (SEM).

Immunohistochemical analysis revealed that ATP13A2 was positively expressed in 80% (24 cases) of tumour tissues, with strong positivity in 50% (15 cases). In contrast, P16 was positive in all cases. Representative IHC images revealed a prevalent ATP13A2 protein expression in cervical tissue (Figure 2A), with notably stronger staining in cancerous tissues than in adjacent non‐malignant epithelia. In contrast, cervicitis samples exhibited lower ATP13A2 staining in epithelial and stromal tissues. ATP13A2 expression levels in epithelial tissues of cervicitis samples were markedly lower than those in cervical squamous epithelial cancer tissues (Figure 2B), indicating a potential differential diagnostic value.

### 3.6 Immune infiltration analyses

**FIGURE 3:**
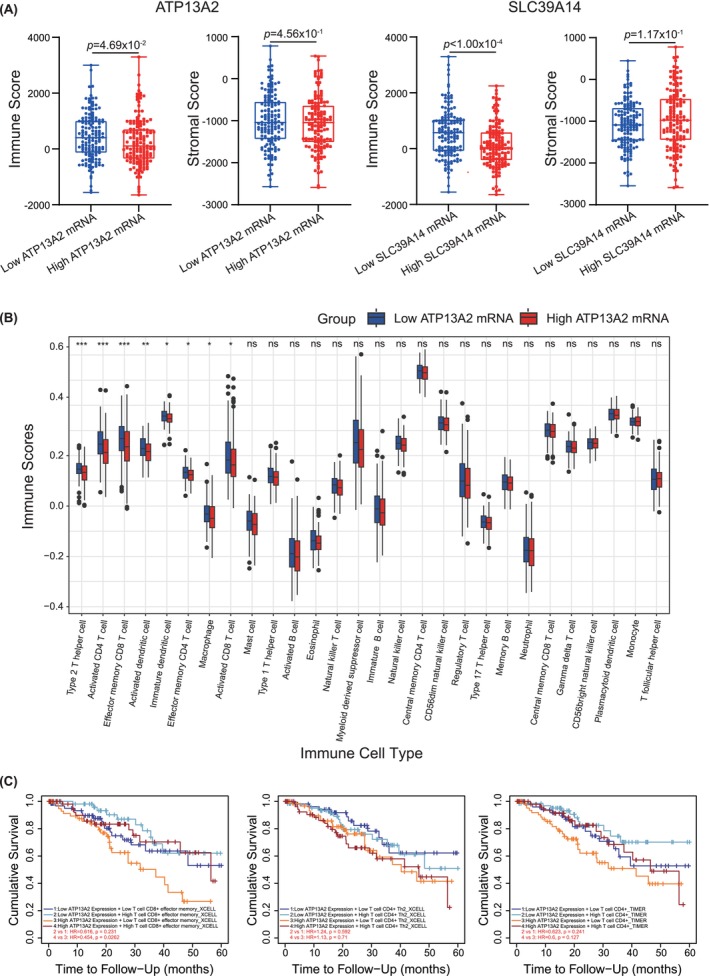
Evaluation of immune infiltration across high and low gene expression groups. (A) Plots show lower immune scores in the high gene expression group than in the low gene expression group for ATP13A2 and SLC39A14. Data are sourced from a Cervical Squamous Cell Carcinoma study (TCGA, PanCancer Atlas), *n* = 147 for the high and low gene expression groups. (B) Comparison of 28 immune cell subtypes between patients with high ATP13A2 mRNA expression level and controls. Histogram showing the differences in infiltration levels of type 2T helper cells, activated CD4 T cells, effector memory CD8 T cells, activated dendritic cells, immature dendritic cells, effector memory CD4 T cells, macrophages and activated CD8 T cells between high and low ATP13A2 expression groups. *p < 0.05; **p < 0.01; ***p < 0.001; ns, not significant. (C) Clinical survival outcomes of CC patients in the effector memory CD8 T cell, type 2T helper cell and activated CD4 T cell groups.

To investigate the relationship between ATP13A2 expression levels and immune cell infiltration in CC, we categorized CC samples from the cBioPortal and ESTIMATE databases into high and low ATP13A2 expression groups. This categorization was also applied to the 31 candidate genes identified in the PPI analysis. The samples in the high‐expression group exhibited significantly lower immune scores, particularly for ATP13A2 and SLC39A14, suggesting a reduced immune cell presence. Conversely, stromal scores showed no significant variation between the high‐ and low‐expression groups, indicating that the stromal component remained unaffected by the expression of these genes (Figure 3A).

To further investigate the relationship between ATP13A2 and immune cells, we used ssGSEA from the R package to investigate the potential association between ATP13A2 expression and 28 types of immune cells. TP13A2 expression levels were significantly negatively correlated with type 2T helper cells, activated CD4^+^ T cells, effector memory CD8^+^ T cells, activated dendritic cells, immature dendritic cells, effector memory CD4^+^ T cells, macrophages and activated CD8^+^ T cells (Figure 3B). Finally, we assessed the impact of immune cell infiltration on clinical survival outcomes in patients with CC using TIMER (http://timer.cistrome.org/). Low levels of effector memory CD8^+^ T cells were associated with poor prognosis (*p* < 0.05; Figure 3C).

### 3.7 Survival prognosis analyses

**FIGURE 4:**
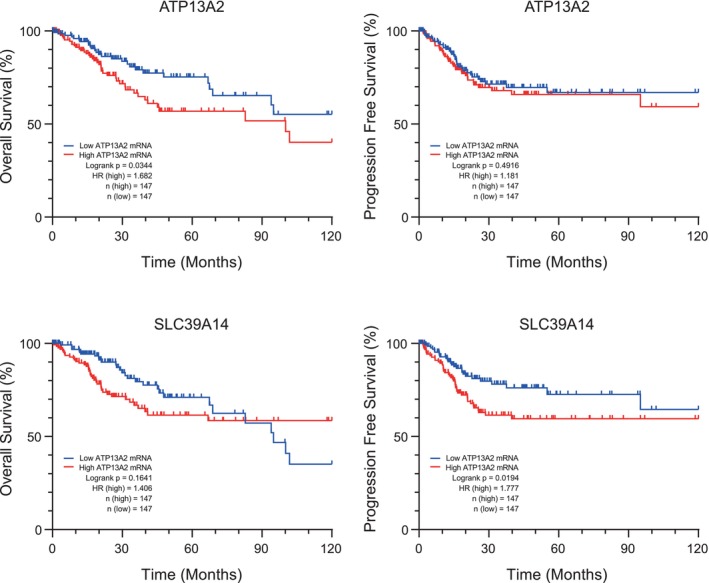
Comparison of overall survival (OS) and progression‐free survival (PFS) between high and low gene expression groups. Kaplan–Meier survival curves representing the OS of patients grouped based on low (blue curve) and high (red curve) ATP13A2 and SLC39A14 expression levels. The *x*‐axis represents survival duration (in months), and the *y*‐axis represents survival probability. Significant differences between the two groups were determined using the log‐rank test. Data are sourced from a Cervical Squamous Cell Carcinoma study (TCGA, PanCancer Atlas), *n* = 147 for the high and low gene expression groups.

To investigate the role of ATP13A2 in CC further, we obtained clinical data from the cBioPortal database and classified patients into high‐ and low‐expression groups for ATP13A2 and SLC39A14. Kaplan–Meier survival curves were constructed to assess the impact of ATP13A2 and its associated genes on patient outcomes. Patients in the high‐expression group had significantly poorer OS than those in the low‐expression group. Additionally, patients with high SLC39A14 expression exhibited markedly reduced PFS, as determined via the log‐rank (Mantel–Cox) test (Figure 4).

### 3.8 Analyses of clinical CC features

**FIGURE 5:**
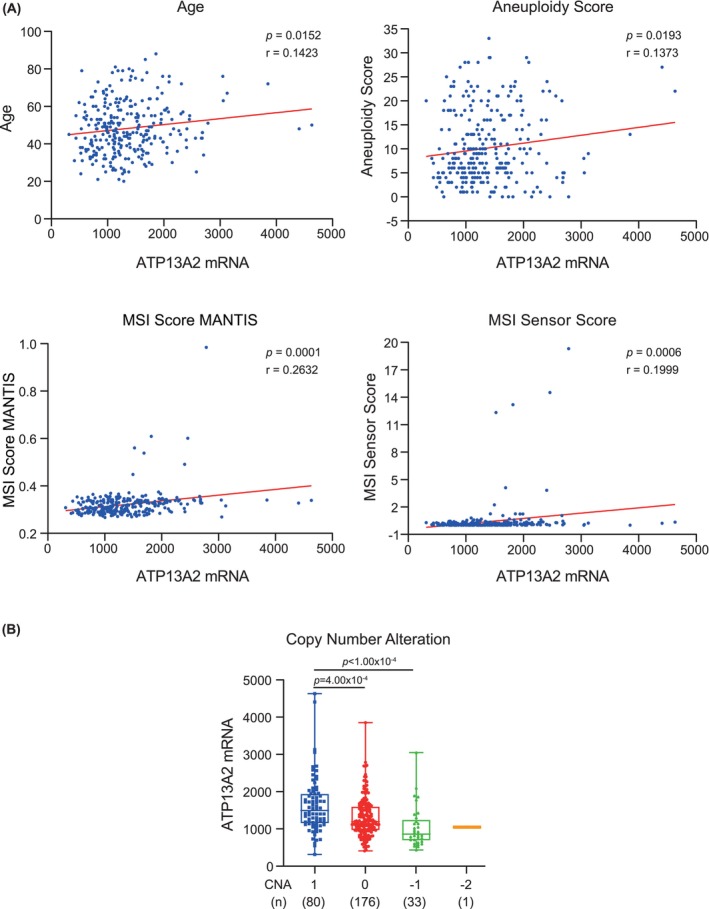
Pearson's correlations between ATP13A2 expression levels and various clinical features in cervical cancer. Data are sourced from a Cervical Squamous Cell Carcinoma study (TCGA, PanCancer Atlas), with 290 available cases for analysis. (A) Scatter plots display the correlation between ATP13A2 expression levels (x‐axis) and various clinical features (y‐axis), including age, aneuploidy score, MSI score MANTIS and MSI sensor score. Each dot represents an individual patient's data. Linear regression lines (solid red lines) indicate the direction and strength of the correlations. Pearson's correlation coefficient (*r*) and significance level (*p*‐value) are displayed for each plot. (B) ATP13A2 expression in cervical cancer stratified by copy number alterations (CNAs). The box plot represents the distribution of ATP13A2 expression levels (y‐axis) in cervical cancer samples grouped by their respective CNAs (x‐axis): CNA = 1, 0, −1 and −2. Each box represents the full range of minimum to maximum expression values. All data points are displayed within the box. The mean ATP13A2 expression is highlighted by a horizontal line within a box. The number of samples in each CNA group is indicated by the corresponding box. CNA = 1, gain of copy number; CNA = 0, neutral or no change in copy number; CNA = −1, single copy deletion; CNA = −2, double copy deletion.

To further investigate the correlation between ATP13A2 expression levels and clinical features in CC, we conducted correlation analyses involving various clinical features, including age, aneuploidy score and MANTIS and MSI sensor scores for microsatellite instability. The results indicated a positive correlation between ATP13A2 expression levels and age, aneuploidy score, MSI score MANTIS and MSI sensor score (Figure 5A). These results suggest that higher expression of ATP13A2 was more prevalent in older patients with CC.

Furthermore, we analysed the relationship between ATP13A2 protein abundance (as determined by IHC staining results; Figure 2A) and patient age. Consistent with our hypothesis, elevated ATP13A2 abundance was predominantly observed in the 50–69 years group (Table 2). Additionally, ATP13A2 was highly expressed in patients with many CNAs (Figure 5B).

**TABLE 2:** Age.

Age	ATP13A2 histological scores				Total
	Negative (0)	Weak (1)	Moderate (2)	Strong (3)	
0–49	1 (3.3%)	3 (10%)	1 (3.3%)	2 (6.7%)	7
50–59	5 (16.7%)	2 (6.7%)	1 (3.3%)	12 (40%)	20
60–69	0 (0%)	0 (0%)	2 (6.7%)	1 (3.3%)	3

*Note*: *χ* = 13.53, *p* < 0.05.

### 3.9 Network correlation of bacterial infiltration and clinical features

To elucidate the association between bacterial abundance in CC tissues and clinical traits, we utilized TCGA data sets to create a network model of bacterial abundance and clinical features using the ‘WGCNA’ package in R. We categorized 1406 microorganisms into six distinct clusters. Subsequently, we computed the average linkage. Pearson's correlation coefficients were used in cluster analysis across all data set samples to investigate the interplay between tumour bacterial abundance and clinical characteristics.

We established a scale‐free network, selecting a soft thresholding power of β = 2. This analytical approach identified six modules associated with ATP13A2 mRNA expression (Figure S1A). The turquoise module exhibited the most substantial correlation with ATP13A2 mRNA (Figure S1B; r = 0.81, *p* = 2.6e‐188) and was consequently designated the central hub module. This module highlighted microbial taxa with high connectivity, potentially playing a critical role in ATP13A2 mRNA expression levels. We extracted the top 10 microbial taxa from this module, ranked by their Pearson's correlation coefficients, for further consideration, including *Senegalimassilia* and *Magnetospira* (Figure S1C) among others (Figure S2).

## 4 DISCUSSION

We initially identified ATP13A2 as a novel gene for predicting clinical prognosis in CC by analysing gene expression differences between CC and normal tissues in TCGA and GEO databases in this study. Moreover, we selected 31 differential genes associated with ATP13A2 in CC using PPI analysis to explore their potential roles. Patients with elevated ATP13A2 expression exhibited significantly lower immune scores. Although the invasion score has not been definitively shown to have a clear relationship with tumour survival prognosis, low immune cell infiltration has been associated with poor prognosis.^23^, ^24^ These observations suggest that ATP13A2 can regulate the TIME, and its high expression is associated with a poor prognosis. Prognostic and immune infiltration analyses of SLC39A14 in related genes yielded similar results. SLC39A14, also called zip‐14, belongs to the SLC39A transmembrane metal transporter family;^25^ its expression is upregulated in gastric and renal cancer tissues, making it a potential prognostic biomarker.^26^, ^27^

Additionally, through GO and KEGG enrichment analyses, along with a review of the existing literature, we discovered that ATP13A2 may attain oncogenic activity in CC. KEGG and GO enrichment analyses were used to elucidate the mechanism(s) by which ATP13A2 and its associated genes impact CC onset and progression. ATP13A2 and its related genes are involved in metal ion pathways, such as iron and calcium ion signalling pathways. ATP13A2 also impacts other diseases by affecting ion channel synthesis. For example, Tillinghast et al. revealed that ATP13A2 exerts a neuroprotective effect by encoding spermine ion channel protein.^28^ Given the association between SLC39A14 and ATP13A2 and the similarities in immune and prognostic profiles, we hypothesize that ATP13A2 may promote tumour progression through an ion channel mechanism similar to SLC39A14, which may cooperate with other genes. However, the specific mechanism requires further investigation.

Next, we confirmed that ATP13A2 expression correlated with immune cell infiltration. A significant negative correlation was observed between ATP13A2 expression and type 2T helper cells, activated CD4^+^T cells and activated CD8^+^ T cells. Furthermore, we found that decreased levels of CD8^+^ T cells were correlated with poor prognosis. CD8^+^ T cells are important components of the TME and have complex roles in cancer progression and prognosis.^29^ The prevention of CD8^+^ T cell dysfunction can serve as a potential strategy for treating malignancies.^30^, ^31^ A previous study identified CD8^+^ T cell infiltration as an independent beneficial factor in CC prognosis.^32^ Moreover, ATP13A2 is associated with CD8^+^ T cells and promotes tumour growth in HCC.^17^ The regulation of the TME is highly complex, and other immune cell types, including dendritic cells and macrophages, may affect patient survival. Hence, further studies are needed to explore the relationship between ATP13A2 expression and such cells.

The IHC results further confirmed the increased abundance of ATP13A2 in most CC samples. Clinical analysis showed that most tumours were positive for both ATP13A2 and P16. As a tumour suppressor gene, P16 is a marker for HPV carcinogenesis, and its expression is directly involved in the negative regulation of cell proliferation. In clinical practice, P16 positivity serves as a marker for the early diagnosis of HPV‐related CC.^33^ Notably, the expression level of ATP13A2 in CC exhibited an age‐related pattern, which coincided with the high incidence of CC.^34^

In summary, our data highlight the potential of ATP13A2 as a reliable diagnostic and prognostic marker of CC. However, due to the limited sample size, we did not compare clinical data between the high and low ATP13A2 protein expression groups. Previous studies, such as those conducted by Chen et al., have shown that patients with high ATP13A2 expression have significantly reduced OS compared to those with lower expression levels.^35^ Our analysis of CC using TCGA database corroborated this finding. However, further studies with larger sample sizes and stratified analyses are required.

High ATP13A2 expression in CC patients was significantly associated with advanced age, high aneuploidy score, high MSI score MANTIS, high MSI sensor score, and high CNAs. In recent studies, the aneuploidy score has emerged as an important marker for prognostic analysis, with higher scores indicating worse survival outcomes. For instance, Verschoor et al. noted that patients with HER2‐negative metastatic breast cancer and elevated aneuploidy scores had shorter PFS and OS.^36^ MSI is an important tool for identifying gynaecological malignancies and predicting treatment outcomes. According to Noh et al., high MSI (MSI‐H) is most frequently observed in endometrial/uterine cancers, followed by ovarian, tubal‐peritoneal and CC.^37^ Moreover, Wong et al. compared cervical squamous cell carcinoma tissues with cervicitis tissues and found a predominance of MSI positivity in the former, with a significantly less favourable prognosis for MSI‐positive patients than patients with microsatellite stability.^38^ The MANTIS score was utilized to predict the MSI status of patients. The higher the score, the more likely the cancer tissue has an MSI‐H status. The results showed that ATP13A2 expression positively correlated with the MSI score MANTIS and MSI scores. CNAs are important cancer cell biomarkers, playing an important role in malignant changes in colonic tissues^39^, ^40^ and the activation of oncogenes and inactivation of tumour suppressors. Lu et al. found that a lower CNA burden was associated with improved clinical benefits and OS.^41^ Significantly more samples with high ATP13A2 expression levels had an increased CNA burden. In summary, elevated ATP13A2 expression is a significant indicator for the identification of CC and predicting adverse prognostic outcomes.

No previous studies have focused on the intratumoral microbiota in CC, providing novel insights into the bacterial communities prevalent under high ATP13A2 expression conditions within the TME. Hence, we delved further into the relationship between high ATP13A2 expression in CC tissues and bacterial infiltration into the TME. Ten candidate microbial taxa, namely *Senegalimassilia*, *Magnetospira*, *Mastigocladopsis*, *Candidatus solibacter*, *Sandarakinorhabdus*, *Halobacterium*, *Tanticharoenia*, *Nyavirus*, *Thermomicrobium* and *Nitritalea* were identified as being the most highly associated with ATP13A2 expression. The tumour microbiome exhibits substantial inter‐individual heterogeneity due to host influences, including age, living environment, genetic factors and habits. The composition of the tumour microbiome undergoes similar changes during cancer progression and treatment, such as the evolution of the gut microbiome during CRC treatment.^42^ Whether intratumoral microbiome changes have similar manifestations in CC remains unclear. Recent studies have focused on the relationship between the cervicovaginal microbiome (CVM) and the occurrence and progression of CC.^43^, ^44^, ^45^ These studies have revealed vaginal bacterial colonization with *Gardnerella*, *Prevotella*, *Atopobium* and *Megasphaera* in over 40% of persistent HPV‐positive women in the community state type IV subgroup.^46^, ^47^ It has been hypothesized that the dynamic interaction between HPV and CVM creates an unbalanced cervicovaginal microenvironment, triggers biological dysregulation, enhances HPV persistence and promotes CC. The intratumoral microbiota originates from the bacterial translocation of the associated tissues, such as intratumoral *Streptococcus* in oesophageal carcinoma, presumably caused by bacterial translocation from the gut.^48^ However, the candidate microbial taxa from our research were primarily associated with the gut microbiota, which differs from existing studies on CVM. The theory of CC tumour bacterial infiltration has yet to be established, and the source of invasive bacteria in the TME remains controversial, warranting further investigation. Our study did not explore whether bacterial groups and ATP13A2 promote tumorigenesis and progression. According to a previous gene enrichment analysis, the role of microbiota and ATP13A2 in promoting tumour progression may be regulated by transcription. However, the analysis of tumour bacterial groups obtained in this study can provide a basis for further exploration of the connection between the bacterial microenvironment and the TME.

With the elucidation of ATP13A2 expression in CC, survival analysis, associations with clinicopathological factors, co‐expression networks, gene set enrichment analysis, and crosstalk with immune infiltration, ATP13A2 has been suggested as a potential novel prognostic and diagnostic biomarker for CC. Furthermore, it may be a promising candidate for targeted treatment of CC. However, our study has certain limitations. Although we used bioinformatics and experimental validation to explore the relationship between ATP13A2 expression and CC prognosis, the specific mechanisms by which ATP13A2 influences CC progression remain unclear. Furthermore, our investigation into tumour bacterial infiltration is preliminary, identifying the primary bacterial population without further exploring the source of these bacteria or their precise role within the tumour. This underscores the need for more comprehensive experimental data to validate these initial findings.

## 5 CONCLUSION

Our results suggest that ATP13A2 is a promising diagnostic and prognostic marker for CC. Nevertheless, further large‐scale studies are essential to fully comprehend the mechanisms underlying the involvement of ATP13A2 in CC. The complex interactions between ATP13A2 and numerous molecular pathways, especially their correlation with specific clinical characteristics, pave the way for novel opportunities in tailoring treatment approaches and fostering additional research in the context of CC.

## AUTHOR CONTRIBUTIONS

**Zhi Zhao:** Conceptualization (equal); funding acquisition (equal); project administration (equal); supervision (equal). **Yijie Peng:** Data curation (equal); methodology (equal); writing – original draft (equal); writing – review and editing (equal). **Yuanyuan Yang:** Investigation (equal); resources (equal). **Shuaiyu Li:** Visualization (equal). **Jiang Ling:** Data curation (equal); resources (equal). **Zhenyu Zhu:** Conceptualization (equal); investigation (equal); project administration (equal); validation (equal); visualization (equal). **Chenfeng He:** Conceptualization (equal); data curation (equal); formal analysis (equal); investigation (equal); methodology (equal); visualization (equal); writing – review and editing (equal).

## FUNDING INFORMATION

This study was funded by the Henan Province Medical Science and Technology Research Project (LHGJ20210801) and a scholarship from the China Scholarship Council under grant number 202108410243 (CH). The funders were not involved in the study design, collection, analysis and interpretation of data, writing of this article or decision to submit it for publication.

## CONFLICT OF INTEREST STATEMENT

The authors declare no conflicts of interest.

## CONSENT

Not applicable.

## Supporting information


**FIGURE S1.** Network correlation of bacterial abundance and clinical features based on WGCNA. (A) Microbial taxa abundance network constructed using the R package ‘WGCNA’ based on data from TCGA data set. A total of 1406 microorganisms were grouped into six distinct clusters using the dynamic tree‐cut method. This analysis elucidated the relationship between bacterial abundance within tumours and various characteristics, including ATP13A2 expression levels and other clinical features. The heat map presents bacterial taxa modules that are either positively (red) or negatively (green) correlated with clinical features. (B) The scatter plot displays the correlation between module membership values and microbial taxon abundance within the most significant module (turquoise module) linked to ATP13A2 expression. Each point on the plot represents a specific microbial taxon. (C) Scatter plots illustrate the correlation between ATP13A2 expression and the top 10 microbial taxa from the most significant module of cervical cancer patients. Each point on the plot represents an individual clinical data point. The best‐fit line provides a visual representation of the overall trend. The figure shows the top 2 microbial taxa, *Senegalimassilia* (*p* < 0.0001, *r* = 0.3445) and *Magnetospira* (*p* < 0.0001, *r* = 0.3444), as two prominent microbial taxa from the turquoise module, ranked by Pearson’s correlation coefficient. An additional eight microbial taxa are shown in more detail (see Figure S2).
**Figure S2.** Correlation between ATP13A2 expression and microbial taxa abundance in cervical cancer. Scatter plots illustrating the correlation between ATP13A2 expression and the additional eight microbial taxa from the most significant module in cervical cancer patients. Each point on the plot represents individual clinical data. These microbial taxa are part of the top 10 from the turquoise module and were selected based on their significance in relation to ATP13A2 expression, following *Senegalimassilia* and *Magnetospira* (see Figure S1C).

## Data Availability

Data supporting this article are available in the Methods section of the manuscript or can be obtained by contacting the authors.
