# Supplementary figures and images for: ATP13A2 as a prognostic biomarker and its correlation with immune infiltration in cervical cancer: A retrospective study
Source: J Cell Mol Med. 2025 Apr 8;29(7):e70097. doi: 10.1111/jcmm.70097 (PMC11976316; doi:10.1111/jcmm.70097)

Figure S1

(A)

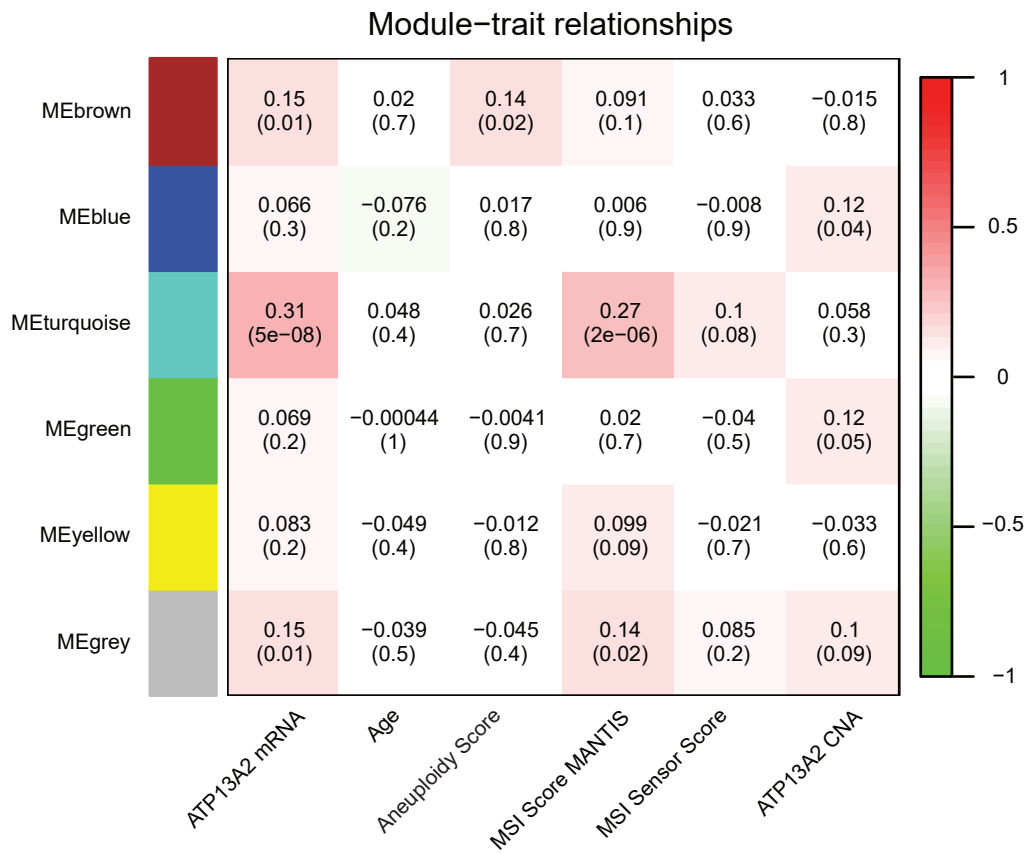

(B)

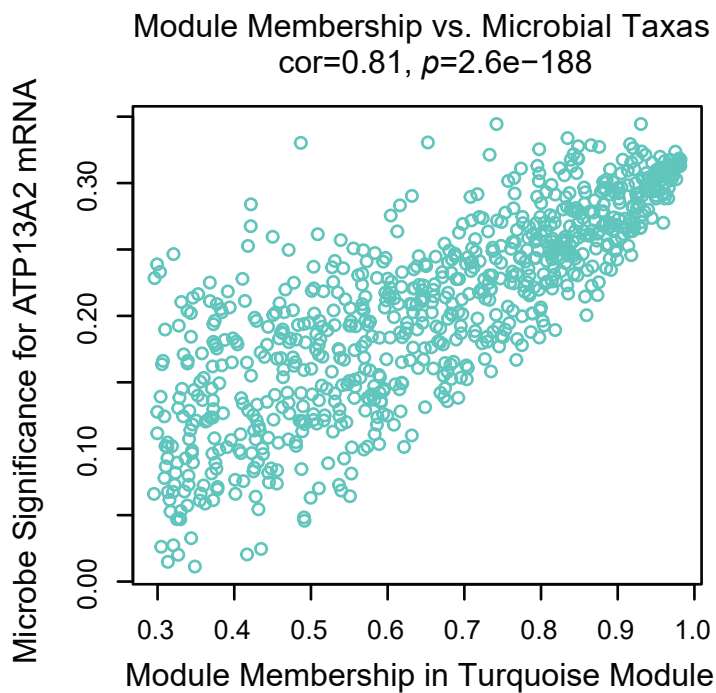

(C)

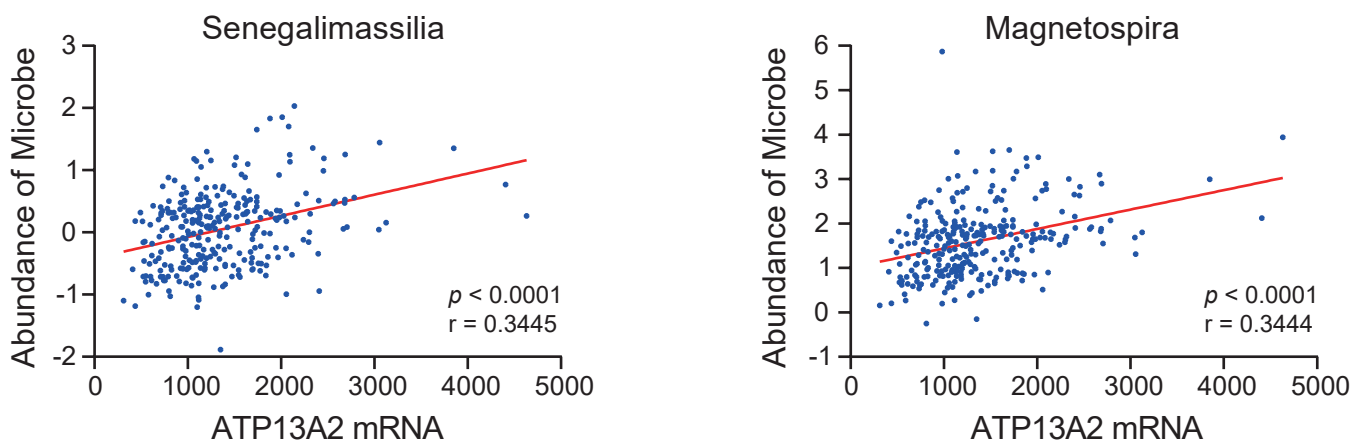

Figure S2

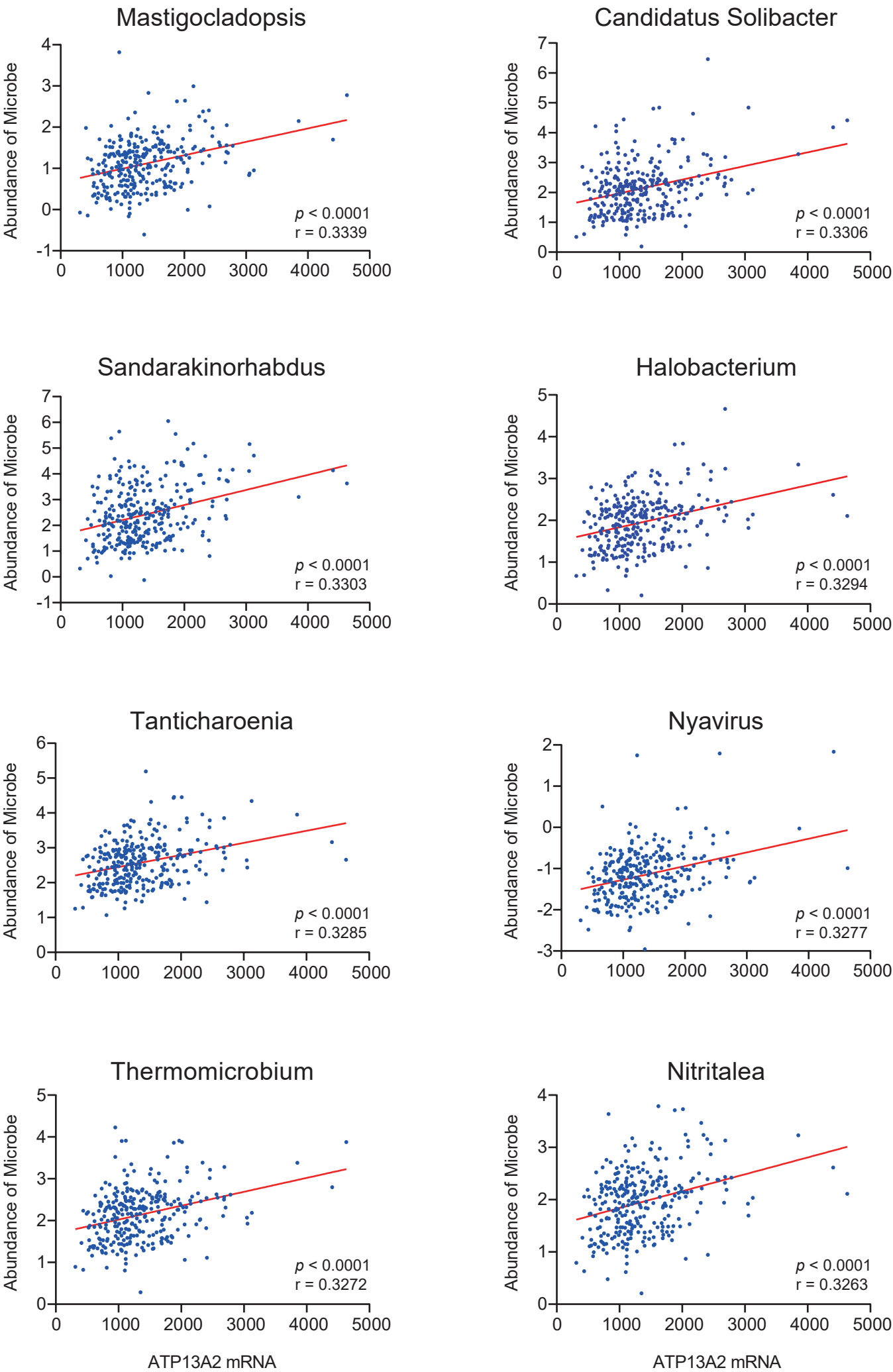

Supplement: Supplementary file 1 — FIGURE S1. Network correlation of bacterial abundance and clinical features based on WGCNA. (A) Microbial taxa abundance network constructed using the R package ‘WGCNA’ based on data from TCGA data set. A total of 1406 microorganisms were grouped into six distinct clusters using the dynamic tree‐cut method. This analysis elucidated the relationship between bacterial abundance within tumours and various characteristics, including ATP13A2 expression levels and other clinical features. The heat map presents bacterial taxa modules that are either positively (red) or negatively (green) correlated with clinical features. (B) The scatter plot displays the correlation between module membership values and microbial taxon abundance within the most significant module (turquoise module) linked to ATP13A2 expression. Each point on the plot represents a specific microbial taxon. (C) Scatter plots illustrate the correlation between ATP13A2 expression and the top 10 microbial taxa from the most significant module of cervical cancer patients. Each point on the plot represents an individual clinical data point. The best‐fit line provides a visual representation of the overall trend. The figure shows the top 2 microbial taxa, Senegalimassilia (p < 0.0001, r = 0.3445) and Magnetospira (p < 0.0001, r = 0.3444), as two prominent microbial taxa from the turquoise module, ranked by Pearson’s correlation coefficient. An additional eight microbial taxa are shown in more detail (see Figure S2). Figure S2. Correlation between ATP13A2 expression and microbial taxa abundance in cervical cancer. Scatter plots illustrating the correlation between ATP13A2 expression and the additional eight microbial taxa from the most significant module in cervical cancer patients. Each point on the plot represents individual clinical data. These microbial taxa are part of the top 10 from the turquoise module and were selected based on their significance in relation to ATP13A2 expression, following Senegalimassili [file JCMM-29-e70097-s001.pdf]
